# Invasive Rhinosinusitis Caused by *Alternaria infectoria* in a Patient with Autosomal Recessive CARD9 Deficiency and a Review of the Literature

**DOI:** 10.3390/jof8050446

**Published:** 2022-04-25

**Authors:** Olivier Paccoud, Nicolas Vignier, Mohammed Boui, Mélanie Migaud, Pierre Vironneau, Romain Kania, Frédéric Méchaï, Sophie Brun, Alexandre Alanio, Arnault Tauziède-Espariat, Homa Adle-Biassette, Elise Ouedraogo, Jacinta Bustamante, Olivier Bouchaud, Jean-Laurent Casanova, Anne Puel, Fanny Lanternier

**Affiliations:** 1 Université de Paris, Necker-Pasteur Center for Infectious Diseases and Tropical Medicine, Necker-Enfants Malades University Hospital, Assistance Publique des Hôpitaux de Paris (AP-HP), 75015 Paris, France; opaccoud@hotmail.com; 2 Université Sorbonne Paris Nord, IAME, INSERM UMR 1137, Department of Infectious and Tropical Diseases, Avicenne University Hospital, AP-HP, Hôpitaux Universitaires Paris Seine-Saint-Denis, 93000 Bobigny, France; dr.vignier@gmail.com (N.V.); frederic.mechai@aphp.fr (F.M.); elise.ouedraogo@aphp.fr (E.O.); olivier.bouchaud@aphp.fr (O.B.); 3 Centre d’Investigation Clinique Antilles Guyane, Inserm CIC 1424, Centre Hospitalier de Cayenne, Cayenne 97306, French Guiana; 4 Department of Dermatology, Military Hospital Mohamed V, Rabat 10045, Morocco; m.boui@um5r.ac.ma; 5 Laboratory of Human Genetics of Infectious Diseases, Necker Branch, INSERM U1163, 75015 Paris, France; melanie.migaud@inserm.fr (M.M.); casanova@mail.rockefeller.edu (J.-L.C.); anne.puel@inserm.fr (A.P.); 6 Imagine Institute, Université de Paris, 75015 Paris, France; 7 Department of Otorhinolaryngology, Head and Neck Surgery, Université de Paris, Lariboisière University Hospital, 75010 Paris, France; piervir@yahoo.fr (P.V.); romain.kania@aphp.fr (R.K.); 8 Department of Parasitology and Mycology, Université Sorbonne Paris Nord, Avicenne University Hospital, AP-HP, Hôpitaux Universitaires Paris Seine-Saint-Denis, 93000 Bobigny, France; sophie.brun@aphp.fr; 9 Université de Paris, Laboratoire de Parasitologie-Mycologie, Hôpital Saint-Louis, AP-HP, 75010 Paris, France; alexandre.alanio@pasteur.fr; 10 Department of Pathology, Université de Paris, Lariboisière Hospital, AP-HP, DMU, DREAM, UMR 1141, INSERM, 75010 Paris, France; arnault.tauziedeespariat@gmail.com (A.T.-E.); homa.adle@aphp.fr (H.A.-B.); 11 Study Center for Primary Immunodeficiencies (CEDI), Necker-Enfants Malades University Hospital, AP-HP, 75015 Paris, France; jacinta.bustamante@inserm.fr; 12 St. Giles Laboratory of Human Genetics of Infectious Diseases, The Rockefeller University, New York, NY 10065, USA

**Keywords:** *Alternaria infectoria*, CARD9 deficiency, phaeohyphomycosis, invasive fungal sinusitis

## Abstract

Phaeohyphomycoses comprise a heterogeneous group of fungal infections caused by dematiaceous fungi and have primarily been reported in patients with underlying acquired immunodeficiencies, such as hematological malignancies or solid-organ transplants. Over the past decade, a growing number of patients with phaeohyphomycosis but otherwise healthy were reported with autosomal recessive (AR) CARD9 deficiency. We report a 28-year-old woman who presented with invasive rhinosinusitis caused by *Alternaria infectoria*. Following a candidate gene sequencing approach, we identified a biallelic loss-of-function mutation of CARD9, thereby further broadening the spectrum of invasive fungal diseases found in patients with inherited CARD9 deficiency. In addition, we reviewed 17 other cases of phaeohyphomycosis associated with AR CARD9 deficiency. Physicians should maintain a high degree of suspicion for inborn errors of immunity, namely CARD9 deficiency, when caring for previously healthy patients with phaeohyphomycosis, regardless of age at first presentation.

## 1. Introduction

Phaeohyphomycoses comprise a heterogeneous group of fungal infections caused by dematiaceous, or darkly pigmented fungi, of which over 150 species and 70 genera have been involved in human disease [1]. Their defining characteristic is the presence of melanin in their cell walls, which is considered to be a significant virulence factor [2]. Phaeohyphomycoses are responsible for a wide range of clinical symptoms, which vary according to the disease-causing fungal species, host immune status, and route of infection [3]. These clinical features include allergic disease, onychomycosis, superficial cutaneous or subcutaneous disease, keratitis, invasive sinusitis, brain abscesses, and disseminated disease [3,4]. Phaeohyphomycoses have primarily been reported in patients with underlying immunodeficiencies, such as hematological malignancies or in solid-organ transplantation [5,6,7]. Over the past decade, there have also been a growing number of reports of phaeohyphomycoses occurring in otherwise healthy individuals and revealing loss-of-function mutations of CARD9 [8].

Among dematiaceous fungi, the genus *Alternaria* comprises over 80 species, with *A. alternata* and *A. infectoria* being responsible for most cases of human disease [5]. *Alternaria* species have a worldwide distribution, many of them being common saprophytes of soil, air, or agents of decay and plant pathogens. *Alternaria* is most frequently associated with chronic allergic pulmonary or sinus disease [9,10], but it is increasingly reported as a pathogen causing cutaneous and subcutaneous disease, oculomycosis, and rhinosinusitis in immunocompromised hosts, most notably in solid-organ transplant recipients and in patients with hematological malignancy [5,6,7,11]. We studied the case of a 28-year-old woman who presented with invasive rhinosinusitis caused by *A. infectoria*. In addition, we reviewed the literature for cases of phaeohyphomycosis occurring in otherwise healthy individuals, as well as reports of invasive rhinosinusitis caused by *Alternaria* species.

## 2. Materials and Methods

### 2.1. Mutation Analyses

Genomic DNA was isolated from whole blood using the iPrep™ technology (from Invitrogen, Waltham, MA, USA). CARD9 was amplified with specific primers, as previously described [12], using DreamTaq™ green polymerase (Thermofisher scientifics, Waltham, MA, USA). PCR products were analyzed by electrophoresis in 1% agarose gels, sequenced with the Big Dye Terminator V3.1™ cycle sequencing kit (Thermofisher scientifics), and analyzed on a 3500XL Genetic analyzer (Applied Biosystems, Foster City, CA, USA).

### 2.2. Functional Analysis: Whole Blood and PBMCs Stimulation

Peripheral Blood Mononuclear cells (PBMCs) from a patient or healthy donor (n = 1) were isolated from heparin blood samples using Ficoll-Plaque Plus (GE Heathcare, Chicago, IL, USA) according to the kit manufacturer’s instructions, as previously described [13]. Whole blood samples (250 µL) or PBMCs (1 × 10^6^/mL) were set in a 48 wells plates for a final volume of 500 µL in RPMI 1640 (GIBCO, Waltham, MA, USA). They remain unstimulated or stimulated by Curdlan (100 µg/mL; InvivoGen, Toulouse, France), Zymosan (5 µg/mL; InvivoGen), heat-killed *Candida albicans* (HKCA) (10^6^ particles; InvivoGen), heat-killed *Saccharomyces cerevisiae*–HKSC (10^6^ particles; InvivoGen), heat-killed *Exophiala dermatitidis* (10^6^ particles; Pasteur institute, Paris, France), heat-killed *Staphylococcus aureus* (HKSA) (10^6^ particles; InvivoGen), vesicular stomatitis virus—VSV (10^6^ particles), Bacille Calmette Guérin (BCG), Lipopolysaccharide (LPS) (10 µg/mL; InvivoGen) or Phorbol myristate acetate (PMA)/ionomycine (40 ng/mL—2 × 10^−4^; InvivoGen). Supernatants were recovered after 24 and 48 h. IL-6 production in supernatant was measured using a two-sided sandwich ELISA, according to the kit manufacturer’s instructions, as previously described [14], (PeliPair human cytokine Elisa reagent set M9316, Sanquin, Amsterdam, The Netherlands).

### 2.3. Literature Review

We performed a comprehensive review of the literature using Pubmed (with the following search terms: ‘CARD9’, ‘Caspase recruitment domain-containing protein 9′, ‘caspase recruitment domain protein 9’, and ‘caspase recruitment domain family member 9′ AND ‘phaeohyphomycosis’, ‘*Alternaria*’, ‘*Exophiala*’, ‘*Phialophora*’, ‘*Corynespora*’, ‘*Aureobasidium*’, ‘*Pallidocercospora*’, *and* ‘*Ochroconis*’) for all published cases of phaeohyphomycosis occurring in patients with autosomal recessive CARD9 deficiency. All relevant articles published from January 2009 to January 2022 in English or in French were screened. As previously described [6], we considered cases of phaeohyphomycosis to be ‘local superficial’ if they involved only the skin and subcutaneous tissues. We considered cases to be ‘local deep infections’ if they were localized to deep tissues such as the sinus, eyes, lungs, bones, or joints. Finally, infections were classified as ‘disseminated’ in cases of fungemia, or if they involved the central nervous system or at least two non-contiguous sites [6].

In addition, we reviewed the literature for all published cases of invasive rhinosinusitis caused by *Alternaria* spp. from 1977 to January 2022. We excluded cases of noninvasive sinusitis, defined as cases of sinusitis without invasion of the mucosa, blood vessels, or bone, i.e., all cases of allergic fungal sinusitis and sinus mycetoma [15,16]. We also excluded cases found in case series of phaeohyphomycoses or invasive fungal sinusitis in which data from individual patients were not available in the manuscript and/or Supplementary Materials [6,7,17,18,19].

## 3. Results

### 3.1. Invasive Rhinosinusitis Caused by Alternaria Infectoria in an Otherwise Healthy Woman

A 28-year-old Moroccan woman with no significant medical history was referred to the Infectious Diseases Department of Avicenne Hospital, Bobigny (Paris area), France, with a suspicion of invasive fungal rhinosinusitis. She resided and worked on a farm near Rabat, Morocco, where she tended cattle. During her first pregnancy in Morocco at age 23, she presented a non-healing ulceration of the right ankle without notion of prior trauma. Skin biopsies showed an altered tissue organization with inflammatory infiltrates and multiple spherical hyphae, but mycological cultures exhibited no growth. Due to a suspicion of cutaneous mucormycosis, she received a six-week course of liposomal amphotericin B (L-AmB), followed by surgical excision of the lesion and subsequent skin engraftment. During a second pregnancy at age 25, she experienced epistaxis and complained of a low-grade fever and headache. Physical examination revealed hard-palate necrosis with oronasal communication. Due to the suspicion of relapse of an invasive fungal infection, she received a second 3-month course of L-AmB and was given a palatal prosthesis. Over the ensuing year, she experienced persistent fever and a 22 kg weight loss.

**Figure 1:**
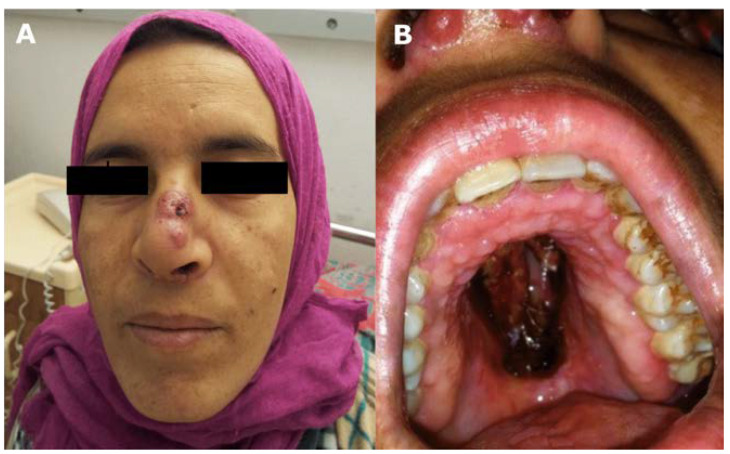
Painful erythematous lesions of the *dorsum nasi* (**A**) and necrotic lesions of the hard palate (**B**) due to *Alternaria infectoria* in a patient with CARD9 deficiency.

After moving to France at age 28, she was referred for a diagnostic workup. At admission, the patient was afebrile. Physical examination revealed painful erythematous lesions of the dorsum nasi, hemorrhagic and crusting lesions of the hard palate, and nasal septum destruction (Figure 1, Panels A and B). Facial computed tomography (CT) and magnetic resonance imaging (MRI), which are featured in a previous report [20], showed a pseudo-tumoral thickening of the left maxillary sinus, as well as lysis of the orbital plate, the perpendicular plate of the ethmoid bone, and the vomer. Cerebral imaging was unremarkable, and a full-body CT scan showed no other localization of disease. Histological analyses of skin and mucosal tissue biopsies (with hematoxylin and eosin stain and Grocott methenamine silver) revealed Grocott-positive large-caliber hyphae with a surrounding gigantocellular macrophagic reaction, but DNA extractions for fungal identification were unsuccessful. Due to a suspicion of invasive rhinosinusal mucormycosis, she was started on L-AmB (5 mg/kg/day) and underwent extensive surgical debridement and nasal amputation. Histological analyses of nasal tissue revealed pronounced inflammation associated with fungal hyphae infiltrating cartilaginous and osseous tissue (Supplementary Figure S1).

Mycological cultures yielded growth of *Alternaria* spp. Antifungal treatment was switched to itraconazole (400 mg/day), and she subsequently underwent reconstructive nasal surgery. After one year of antifungal treatment and more than 8 years of follow-up, the patient did not experience any relapse of the infection.

### 3.2. Fungal Identification

Culture was performed on slants of Sabouraud dextrose agar with gentamicine and chloramphenicol and incubated for 3 weeks at 30 °C. The culture yielded growth of a colony of *A. infectoria*, which was confirmed morphologically and with the sequencing of the ITS locus (ITS1 ITS4 primers): 852 base pair with 100% similarity with strain CNRMA14.282, CNRMA14.13 (with a 100% overlap) using Pasteur Fungibank and with CNRMA10.1081 (among others) using the mycobank ITS database. The sequence was deposited on GenBank under the accession number: ON100876.

### 3.3. Immunological and Genetic Findings

At admission, blood counts showed hemoglobin levels at 10.1 g/dL (reference range 13–17.5 g/dL), leukocytes at 6.9 × 10^9^ cells/L (reference range 4–10 × 10^9^ cells/L), with neutrophils at 3.7 × 10^9^ cells/L (reference range 1.5–7 × 10^9^ cells/L), lymphocytes at 2.3 × 10^9^ cells/L (reference range 1.4–4 × 10^9^ cells/L), and platelet counts at 319 × 10^9^ cells/L (reference range 150–450 × 10^9^ cells/L). C-reactive protein was 1 mg/L (reference < 5 mg/L), and serum immunoglobulin levels were within normal ranges. HIV serological testing was negative.

**Figure 2:**
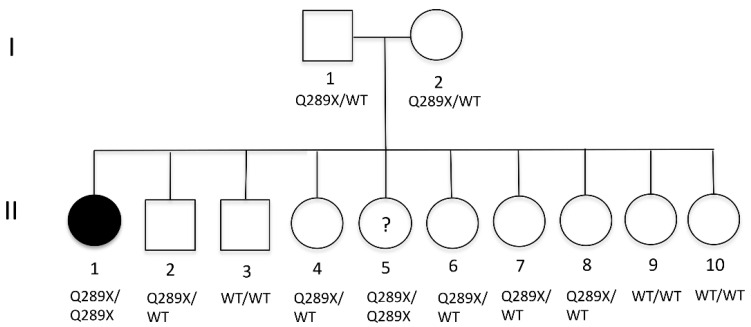
Mutation analyses in the kindred (I: parents, and II: siblings) of the reported patient (patient II.1). WT, wild-type.

**Figure 3:**
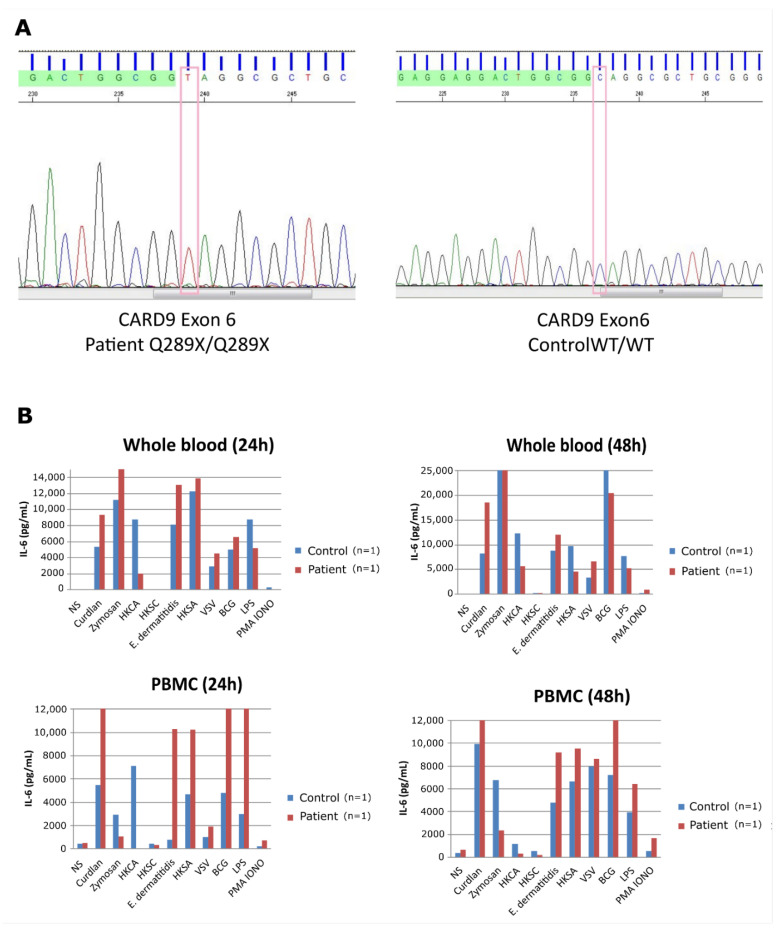
Patient whole blood (**A**) and peripheral blood mononuclear cells (**B**) were stimulated, and interleukin-6 (IL-6) production was measured in the supernatant after 24 and 48 h. Note the subject’s reduced IL-6 production after stimulation with heat-killed *Candida albicans* (HKCA) compared to control. Note. BCG, Bacille Calmette Guérin; LPS, lipopolysaccharide; HKCA, heat-killed *Candida albicans*, HKSC, heat-killed *Saccharomyces cerevisiae*; HKSA, heat-killed *Staphylococcus aureus*; NS, non-stimulated; PMA iono, PMA/ionomycin; VSV, vesicular stomatitis virus.

Due to the invasive nature of the infection and the absence of detectable immunodeficiency after routine immunological explorations, we further investigated the patient. To the best of our knowledge, neither her parents nor her siblings had experienced any significant infectious episodes (Figure 2). History of vaccination with the BCG vaccine or other live-attenuated vaccines was unknown. Using a candidate gene sequencing approach, we sequenced all coding exons of CARD9 and identified a homozygous mutation in exon 6 of CARD9 (c.865C > T), resulting in a premature termination codon at position 289 (p.Q289*) (Figure 3A). Familial segregation showed that both parents (I.1 and I.2), as well as five siblings (II.2, II.4, II.6, II.7, and II.8) were heterozygous for the mutation. Three other siblings (II.3, II.9, and II.19) were wild-type (WT). Interestingly, one of her siblings (II.5) was also homozygous for the same mutation. We were unable to obtain a detailed medical history for this sibling, and she was unfortunately unavailable for further investigations. The allele segregation in the kindred is consistent with AR complete CARD9 deficiency. This c.865C > T mutation has previously been reported in 17 patients originating from North Africa (Algeria, n = 9, Tunisia, n = 4, Morocco, n = 1, and Egypt, n = 1) and South America (Argentina, n = 1 and Colombia, n = 1), all of whom presented with features of invasive fungal infection (extensive or deep dermatophytosis, n = 13, phaeohyphomycosis, n = 2, and *Candida albicans* meningoencephalitis, n = 1) [8,21,22]. Collectively, these data strongly suggest that AR complete CARD9 deficiency caused the invasive *A. infectoria* disease of the patient.

### 3.4. Review of the Literature of Cases of Phaeohyphomycoses Related to AR CAR9 Deficiency

We reviewed the literature for reports of fungal infections occurring in patients with AR CARD9 deficiency and found 17 reports of phaeohyphomycosis in patients originating from China, Germany, Angola, Iran, Colombia, Japan, Argentina, and Morocco [14,21,22,23,24,25,26,27,28,29,30,31]. Patient characteristics, including our case, are summarized in Table 1. Overall, the median age of patients at disease presentation was 21 years (range: 4–48), with a female/male ratio of 1.4. Of these, seven were classified as ‘local superficial’, five as ‘local deep’, and six as ‘disseminated’. *Phialospora* spp. and *Exophiala* spp. were the most commonly involved species, accounting for 10/18 (56%) of the cases. Of note, almost all (5/6, 83%) cases of disseminated diseases were caused by *Exophiala* spp. [14,22,28,29,31]. In contrast, most (4/5) cases of infections caused by *Phialospora* spp. were ‘local superficial’ infections. A notion of prior trauma was reported in only two cases (one patient reported a scissor puncture, and another was wounded by a tree branch [29,30]). Surgery was required for 5/17 cases (data missing for one infection). Of note, 14/18 (77.8%) cases of phaeohyphomycosis, most of which were not disseminated (i.e., the seven ‘local superficial’ infections, and the five ‘local deep’ infections), involved the face, and 11/14 of these included lesions on the cheeks. This is in stark contrast with data from previously published case series of cutaneous/subcutaneous phaeohyphomycosis in immunocompromised hosts (including patients with cancer and hematological malignancy [6,17] and solid-organ transplant recipients [6,11,32,33]), in whom the limbs were the most frequently affected localizations. This possible association between AR CARD9 deficiency and localization of phaeohyphomycosis to the face rather than other exposed body parts remains unexplained. This was particularly evident in patients originating from China (9/10), suggesting a particular environmental factor such as the use of specific cosmetics. However, detailed comparisons of cutaneous localization according to underlying immune status are complicated by the fact that a number of cases of phaeohyphomycosis reported prior to 2009 in apparently immunocompetent patients might have been associated with undiagnosed CARD9 deficiency.

## 4. Discussion

We report a case of phaeohyphomycosis caused by *A. infectoria* in an otherwise healthy 28-year-old woman with invasive rhinosinusitis and AR complete CARD9 deficiency. The case reported here appears to be one of only two published reports of infection caused by *Alternaria* species linked to AR complete CARD9 deficiency [31]. We also found a mention of phaeohyphomycosis caused by *Alternaria tenuis* in the kindred of a previously reported patient with AR CARD9 deficiency, although the specifics of the case were not detailed [22,35]. As cases of infection caused by a wide variety of fungi from the Ascomycota phylum have been reported in association with AR CARD9 deficiency [8], the rarity of prior reports of *Alternaria* infections in this setting most likely reflects underdiagnosis rather than specific susceptibility patterns. This is in part linked to the challenges associated with the mycological diagnosis of phaeohyphomycoses, as mycological cultures are often negative, and molecular identification from fixed tissue is frequently non-contributive.

First described in 2009 in a consanguineous family from Iran with chronic mucocutaneous candidiasis (CMC) and dermatophytosis [36], AR CARD9 deficiency (OMIM: 212050) has since, as of January 2022, been reported in 85 patients (including this reported case) from 62 kindreds [8,21,22,28,31,37,38,39,40,41,42,43,44,45]. CARD9 encodes an adaptor protein, expressed primarily in myeloid cells, which signals downstream from the pattern recognition receptors Dectin-1, Dectin-2, and macrophage-inducible C-type lectin [46]. These receptors recognize pathogen-associated molecular patterns and play a pivotal role in the induction of a pro-inflammatory cytokine cascade, which provides protection against microbial invasion, particularly against pathogenic fungi [47,48,49]. AR complete CARD9 deficiency has been linked to impaired cytokine and chemokine production, ineffective clearance of fungi by neutrophils, and impaired neutrophil recruitment at the sites of infection [8,23]. Age at onset of fungal disease appears to be heterogenous, varying from early childhood to adulthood, including when considering patients with the same fungal disease. 

Of interest, the patient’s leg ulceration appeared during her first pregnancy, and symptoms of rhinosinusitis manifested during a second pregnancy. This is similar to a previous report of a 41-year-old female with phaeohyphomycosis caused by *Exophiala spinifera* and AR CARD9 deficiency (although this was not known at the time of publication), who experienced worsening of cutaneous lesions and generalized lymphadenopathy during pregnancy [35]. We found four additional cases of phaeohyphomycosis in previously healthy young women with worsened disease manifestations at the time of their pregnancy [24,50,51,52]. One possible explanation is that fungal cells may lay dormant in tissues after environmental exposure and may recur due to hormonal changes or immune disbalances during pregnancy [24,53,54].

We reviewed the literature and found 30 other cases of invasive rhinosinusitis caused by *Alternaria* spp., 14 of which were previously reviewed by Pastor and Guarro [5] (including one case which we reclassified as invasive) [5,55,56,57,58,59,60,61,62,63,64,65,66,67,68,69,70,71,72]. Overall, the median age was 26 years (range 2–55 years), with a female/male ratio of 1.3. Twenty patients (66%) were immunocompromised (hematological malignancy or bone marrow transplantation, (n = 19), and acquired immunodeficiency syndrome (n = 1)). The remaining 10 patients were described as immunocompetent, although thorough immunological investigations were not reported. The fact that this review does not include any solid-organ transplant recipients, which are considered to be the most at-risk group of individuals for *Alternaria* infections overall [73], it should be interpreted with caution as a significant number of these were excluded due to the absence of individual patient details in most case series [6,7]. Identification of the fungal species was reported for nine cases only (*A. alternata*, n = 6, *A. infectioria*, n = 2, *A. malorum*, n = 1). Most patients (26/30, 87%) underwent surgical debridement, and all but one received post-operative systemic antifungals, which included amphotericin B in 26/29 (90%) of cases. Four patients, all with hematological malignancy, died during follow-up. Although guidelines for the treatment of phaeohyphomycoses were recently updated, these do not include specific recommendations for Alternaria invasive sinusitis [73]. Due to the rarity of these infections, guidelines are based mostly on anecdotal evidence from case reports/series, and there are no standardized therapies. Species identification and antifungal susceptibility testing should, therefore, always be performed.

## 5. Conclusions

We report a case of invasive rhinosinusitis caused by *A. infectoria* in a previously healthy woman revealing AR CARD9 deficiency. Physicians should maintain a high degree of suspicion for inborn errors of immunity, namely CARD9 deficiency, when caring for previously healthy patients with phaeohyphomycosis, regardless of age at first presentation.

## Figures and Tables

**Table 1:** Literature review of patients with phaeohyphomycosis in the setting of autosomal recessive CARD9 deficiency. 5FC, flucytosine; AmB, amphotericin B; CSP, caspofungin; ITC, itraconazole; F, female; GSV, griseofulvine; L-AmB, lyposomal amphotericin B; m, months; M, male; NA, not available; VRC, voriconazole; TBF, terbinafine; y, year.

N°	Ref	Country	Sex, Age at Presentation	Dissemination	Localization	Species	Trauma	Treatment	Surgery	Mutation	Outcome
1	[23]	China	F, 13	Local Superficial	Skin—face (cheeks) /trunk/limbs	*Exophiala spinifera*	No	TBF + ITC, duration NA	no	Compound: c.C68A → p.S23*; c.819-820insG → p.D274fs*60	Relapse after end of treatment course.
2	[23]	China	F, 45	Local Superficial	Skin—face (cheeks)	*Ochroconis musae*	No	ITC 3m, TBF + ITC 4m, AMB	no	c.819-820insG → p.D274fs*60	Slight improvement in lesions after 7 months of treatment
3	[25]	China	F, 35	Local Deep	Skin—face (forehead, cheeks, nose, mouth)	*Corynespora cassiicola*	No	AMB 3w, lost to follow-up	no	Compound: c.191-192insTGCT → p.L64 fs*59; c.819-820insG → p.D274fs*60	Slight improvement after 2 weeks of treatment, lost to follow-up
4	[34]	China	M, 13	Local Superficial	Skin—face (cheeks)	*Phialophora verrucosa*	No	ITC followed by AMB	no	Compound: c.191-192insTGCT → p.L64 fs*59; c.472C > T → p.Q158*	Worsening and dissemination of lesions despite treatment
5	[34]	China	M, 6	Local Superficial	Skin—face (cheeks)	*Phialophora verrucosa*	No	AMB followed by ITC 2y	no	c.819-820insG → p.D274fs*60	Relapse after treatment cessation
6	[34]	China	F, 20	Local Superficial	Skin—face (cheeks)	*Phialophora verrucosa*	No	ITC 1y	yes	c.819-820insG → p.D274fs*60	Improvement of lesions
7	[34]	China	M, 48	Local Deep	Skin—face (forehead) + endophtalmitis	*Phialophora verrucosa*	No	ITC + TBF 6m	no	c.819-820insG → p.D274fs*60	Slight improvement of lesions after 6 months of treatment
8	[26]	Germany	F, 43	Local Deep	Endophtalmitis	*Aureobasidium pullulans*	No	VRC 3m, VRC 6m	NA	Compound: c.184G > A → p.G62fs*; c.288T > C → p.G96del36	Cured after prolonged antifungal treatment
9	[14]	France	F, 5	Disseminated	Liver + Biliary tract/Brain	*Exophiala dermatidis*	No	L-AMB + VRC 3m followed by VRC 22m	no	c.52C > T → p.R18W	Cured after prolonged antifungal treatment
10	[14]	Iran	F, 18	Disseminated	Skin—limbs/lymph nodes /bones/lungs	*Exophiala spinifera*	No	ITC 3m/ VRC/FCZ (many years)	no	c. GAG967-969del → p.E323del	Progression despite antifungal treatment
11	[21]	Columbia	F, 4	Local deep	Skin—Face (nose, cheeks)/rhinosinus	*Corynespora cassiicola*	No	AmB 1m, VRC + CSP 2w, VRC 12w, multiple relapses	yes	Compound: c.23_29del → p.Asp8Alafs*10; c.865C > T → p.Q289*	Progression despite antifungal treatment and surgical debridement
12	[27]	China	M, 26	Local Superficial	Skin—face (forehead, cheeks)	*Phialophora americana*	No	TBF + ITC, duration NS	no surgery	c.819-820insG → p.D274fs*60	NA
13	[28]	Japan	F, 4	Disseminated	Cerebral/lymph nodes	*Exophiala dermatitidis*	No	VRC 1m, VRC + TBF 2y	no surgery	Compound: c.1118G > C → p.R373P; c.586A > G → p.K196E	Improvement of lesions
14	[22]	Argentina	F, 32	Disseminated	Skin—Face (forehead, cheeks) /limbs/lymph nodes/eyes	*Exophiala spinifera*	No	ITC + 5FC, AmB, L-AmB, GSV, TBF	no surgery	c.865C > T → p.Q289*	Cured after prolonged antifungal treatment
15	[29]	China	M, 23	Disseminated	Skin—face (cheeks)/cerebral/lymph nodes/lungs	*Exophiala dermatitidis*	Yes	L-AmB + VRC 3m followed by VRC 22m	surgery	c.759dup → p.Lys254fs	Died
16	[30]	China	F, 21	Local Superficial	Skin—face (cheeks)	*Pallidocercospora crystallina*	Yes	ITC 5m, ITC + TBF	surgery	c.1118G > C → p.R373P	Cured after antifungal treatment and surgery
17	[31]	China	M, 6	Disseminated	Cerebral	*Alternaria* spp.	No	L-AmB + VRC 4m, VRC 1y	Surgery	Compound: c.G1526A → p.R509K; c.A486G → p.K196E	Improvement after antifungal treatment
18	Present case	Morocco	F, 23	Local Deep	Skin—face (nose) /rhinosinus	*Alternaria infectoria*	No	L-AmB 6w, L-AMB 3m, ITC 1y	surgery	c.865C > T → p.Q289*	Cured after prolonged antifungal treatment and surgery

## Data Availability

Data supporting the case report are available upon request to the corresponding author.

## Supplementary Materials

The following supporting information can be downloaded at: https://www.mdpi.com/article/10.3390/jof8050446/s1, Figure S1: The nose cartilage was invaded by numerous septate hypahea forming branched chains, with catenulate conidia (A: hematoxylin eosin saffron stain, B and C: Grocott, scale bars 50 µm).
