# Supplementary material for: Invasive Rhinosinusitis Caused by Alternaria infectoria in a Patient with Autosomal Recessive CARD9 Deficiency and a Review of the Literature
Source: J Fungi (Basel). 2022 Apr 25;8(5):446. doi: 10.3390/jof8050446 (PMC9144991; doi:10.3390/jof8050446)
Supplement: Supplementary file 1 [file jof-08-00446-s001.zip › jof-1638008-supplementary.pdf]

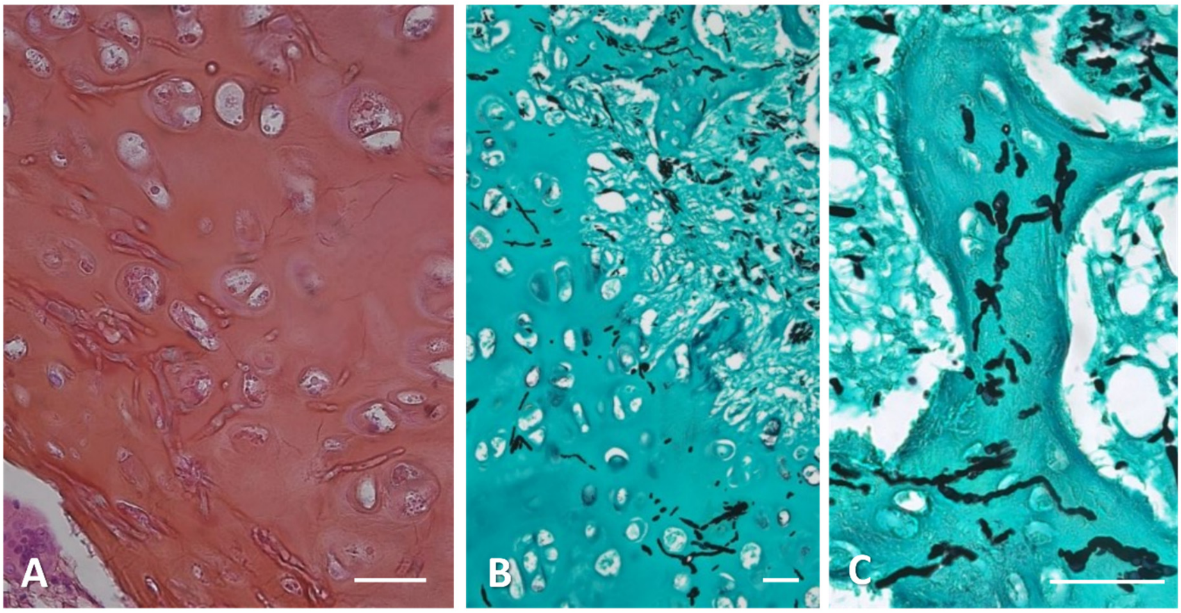

Supplementary Figure S1: The nose cartilage was invaded by numerous septate hypahea forming branched chains, with catenulate conidia (A: hematoxylin eosin saffron stain, B and C: Grocott, scale bars 50µm)
